# Cardiovascular Disease Risk in the Offspring of Diabetic Women: The Impact of the Intrauterine Environment

**DOI:** 10.1155/2012/565160

**Published:** 2012-10-22

**Authors:** Laura J. Marco, Kate McCloskey, Peter J. Vuillermin, David Burgner, Joanne Said, Anne-Louise Ponsonby

**Affiliations:** ^1^Pregnancy Research Centre, The Royal Women's Hospital, Parkville, VIC 3052, Australia; ^2^Murdoch Childrens Research Institute, The Royal Childrens Hospital, Parkville, VIC 3052, Australia; ^3^Child Health Research Unit, Barwon Health, Geelong, VIC 3220, Australia; ^4^Department of Paediatrics, The University of Melbourne, Parkville, VIC 3052, Australia; ^5^Department of Obstetrics and Gynaecology, The University of Melbourne, Parkville, VIC 3052, Australia; ^6^Environmental and Genetic Epidemiology Research Group, Population Health, Genes and Environment Theme, Murdoch Childrens Research Institute, The Royal Childrens Hospital, Parkville, VIC 3052, Australia

## Abstract

The incidence of gestational diabetes is increasing worldwide, exposing large numbers of infants to hyperglycaemia whilst *in utero*. This exposure may have a long-term negative impact on the cardiovascular health of the offspring. Novel methods to assess cardiovascular status in the neonatal period are now available—including measuring arterial intima-media thickness and retinal photography. These measures will allow researchers to assess the relative impact of intrauterine exposures, distinguishing these from genetic or postnatal environmental factors. Understanding the long-term impact of the intrauterine environment should allow the development of more effective health policy and interventions to decrease the future burden of cardiovascular disease. Initiating disease prevention aimed at the developing fetus during the antenatal period may optimise community health outcomes.

## 1. Introduction

The impact of the intrauterine environment on the developing child can be lifelong. Freinkel's theory of fuel-mediated teratogenesis hypothesised that fetal structures may be exquisitely attuned to fine alterations in maternal fuel economy and that these changes could have long-term effects on the metabolic functions of the offspring [1]. 

Approximately 7% of pregnancies are complicated by gestational diabetes mellitus (GDM) (ranging from 1 to 14%, depending on the population studied and the diagnostic tests employed) [2]. Increasing obesity rates mean the proportion of pregnancies complicated by both Type 2 diabetes mellitus (T2DM) and GDM is likely to continue to rise. Exposure to dysglycaemia may have a long-term impact on the developing child's physiology, potentially programming for an increased risk of cardiovascular disease in later life. 

Here we review the early life origins of atherosclerosis and cardiovascular disease. We examine the proposed biological mechanisms linking maternal gestational hyperglycaemia with cardiovascular risk in the offspring of diabetic women. We also review evidence from longitudinal studies of offspring of diabetic women, as well as some of the methodological limitations of these studies. 

We discuss a number of novel methods to assess cardiovascular risk in neonates, which will facilitate improved mechanistic understanding of the effect of *in utero* exposure to diabetes mellitus. These methods focus research efforts on the fetal period as a potential window for future intervention. If further research confirms the importance of intrauterine exposure to hyperglycaemia, independent of genetic and postnatal influences, efforts to prevent cardiovascular disease should shift to include a greater emphasis on interventions during the antenatal period and early childhood to attempt to alter risk trajectories.

## 2. The Early Life Origins of Atherosclerosis and Cardiovascular Disease

Cardiovascular disease remains one of the leading causes of morbidity and mortality globally. In Australia, it is the major cause of death, responsible for 37.6% of all deaths [3]. Whilst cardiovascular diseases usually manifest from middle age onwards, the underlying atherosclerosis develops much earlier and is often present antenatally.

Fatty streaks are present in the vasculature decades before cardiovascular disease becomes symptomatic. Over fifty years ago Holman referred to atherosclerosis as a “pediatric problem” with childhood vascular changes being “that portion of the process below the clinical horizon” [4]. Indeed, current evidence suggests that atherosclerosis and cardiovascular risk begins *in utero* and is compounded by postnatal influences. Autopsy data from fetuses and neonates have found diffuse intima-media thickening in coronary arteries from 36 weeks gestation [5]. Autopsy studies of young people who have died from non-cardiovascular causes have demonstrated that 15–34-year olds almost all have flat fatty streaks in their thoracic and abdominal aortas [6]. Autopsy data from the seminal Bogalusa Heart Study revealed that essentially all persons aged 2–39 had fatty streaks in their aortas [7]. 

## 3. What Mechanisms May Underlie the Link between Maternal Diabetes and Increased Cardiovascular Risk in the Offspring?

Maternal diabetes mellitus increases the risk of pregnancy complications via a myriad of biological mechanisms. Whilst glucose can cross the placenta, insulin cannot; hence diabetes mellitus during pregnancy may expose the fetus to an excessive glucose load. Fetal hyperglycaemia leads to hyperinsulinaemia, causing increased hepatic glucose uptake and glycogen synthesis [8]. This is associated with accelerated fetal weight gain, predominately as increased adiposity [9]. Macrosomic offspring of diabetic women have increased left ventricular mass and increased aortic intima-media thickness compared to controls [10], as discussed subsequently. 

There have been several studies examining the relationship between *in utero *exposure to maternal diabetes and the development of risk factors for cardiovascular disease [10–19]. The underlying biological mechanisms are likely to be multifactorial; these children may be at risk due to metabolic derangements, epigenetic changes, or via a direct effect on their vasculature. 

A number of metabolic derangements have been reported in offspring of diabetic women. Macrosomic offspring of insulin-dependent diabetic women have increased serum lipids, proatherosclerotic apolipoprotein A-I and B-100, and lipoprotein levels at birth when compared to controls [11]. This proatherosclerotic dyslipidaemic state may be due to increased lipid synthesis in the setting of increased substrate availability [20]. These changes may persist into later life predisposing to development of the metabolic syndrome, though longitudinal studies are lacking.

Animal studies demonstrate hyperinsulinism during prenatal development can disrupt the neuroendocrine systems that regulate satiety. Selective inactivation of neuron specific insulin receptor genes in mice results in increased food intake and obesity [21]. Murine models of gestational diabetes demonstrate elevated levels of insulin in the hypothalamus of offspring during perinatal life [22]. In adulthood these mice develop hyperphagia, become overweight, and have impaired glucose tolerance [23, 24]. Perinatal hyperinsulinism in the offspring of diabetic women may potentially increase future risk of offspring overweight by a similar mechanism, though translational data are lacking. A hyperinsulinaemic state during development may alter lifelong hormonal set points, negatively impacting regulators of hunger and metabolism, with a detrimental effect on future cardiovascular health. 

Exposure to hyperglycaemia *in utero* may program future disease risk via changes to critical developmental pathways as a result of altered gene expression. Exposure to maternal diabetes during pregnancy results in significantly altered gene expression in embryos at mid-gestation in a mouse model. Around 1% of the genes surveyed have expression levels that are changed in diabetes-exposed embryos by more than twofold relative to controls. Many of these are transcription factors and DNA-binding molecules known to affect transcriptional regulation. This suggests that altered transcriptional regulation may play a role in the response of embryos to intrauterine exposure to diabetic conditions. Thirty five of these genes were detected in the embryonic cardiovascular system; hence these genes could potentially contribute to cardiovascular pathology [25]. These results have not been replicated in human trials. 

Pax3, a gene required for neural tube closure, is also required for migration of cardiac neural crest from the neural tube to the heart and septation of the primitive cardiac outflow tract into the aorta and pulmonary arteries.The cardiac neural crest also promotes the formation of the smooth muscle component of the tunica media, “middle layer” of the aortic arch and its major branches [26]. Pax3 expression is significantly reduced in the embryos of diabetic mice when compared to embryos of nondiabetic mice. Maternal diabetes inhibits expression of Pax3 in neuroepithelium, possibly through hyperglycemia-induced oxidative stress [27]. This suggests that Pax3 may be an important developmental control gene, expression of which is disturbed in embryos of diabetic mice. These changes could potentially contribute to future adverse cardiovascular effects on the offspring. 

Transient hyperglycaemia has been shown to lead to long-lasting epigenetic changes in primary human and bovine aortic endothelial cells *in vitro*, changes that persist despite subsequent normoglycaemia. This is due to long lasting activating epigenetic changes in the promoter of the nuclear factor *κ*B (NF-*κ*B) subunit p65 in aortic endothelial cells causing increased p65 gene expression [28]. Nuclear factor-*κ*B driven proinflammatory gene expression appears to play a major role in the pathogenesis of atherosclerosis [29]. This data suggests that even short-term intrauterine exposure to hyperglycaemia may have a pervasive effect on the cardiovascular system, contributing to a proinflammatory phenotype in vascular smooth muscle cells. 

Further evidence of epigenetic programming can be seen in the intergenerational effects of gestational diabetes. Mice born to diabetic dams have an increased risk of obesity and spontaneously develop gestational diabetes themselves. The following generation is also at increased risk of impaired glucose tolerance [23]. It is possible that similar mechanisms operate in humans, but shared lifestyle exposures mean the relative impact of intrauterine exposure to maternal diabetes is difficult to quantify. It is known that abnormal nutrition in early life can influence diabetes risk in subsequent generations [30], and exposure to hyperglycaemia due to maternal diabetes mellitus may cause similar effects. 

Offspring of diabetic women exhibit adverse vascular changes that are measurable at birth. These include increased arterial thickness and left ventricular mass [10]. The exact mechanisms underlying these observations are unknown. Similar changes are seen in infants affected by intrauterine growth restriction. Skilton et al. hypothesised these vessel wall changes may result from increased sympathetic tone and intrauterine dyslipidaemia [31]. 

## 4. Outcomes for Offspring of Diabetic Women: Evidence from Longitudinal Studies

Tracking health outcomes for the offspring of diabetic women has been an area of growing research activity and provides important evidence for the long-term effects of exposure to intrauterine hyperglycaemia. 

Studies of the Pima Indian population, who have a high prevalence of T2DM, have been particularly informative. Diabetes during pregnancy is followed by markedly increased rates of offspring obesity at 15–19 years [13], as well as increased rates of T2DM in offspring at age 20–24 years [12]. There is an increased risk of T2DM in offspring of diabetic women when compared to their siblings who were born prior to their mother's diagnosis of diabetes (odds ratio of T2DM 3.7, 95% CI 1.3–11.3) [16]. This suggests that exposure to a hyperglycaemic intrauterine environment has a negative health impact on the offspring above and beyond the effect of other temporally stable characteristics of these mothers, such as genetics or lifestyle choices. However, the Pima Indians have one of the highest rates of T2DM in the world [32], and hence their results may not be generalisable to other populations. 

A prospective cohort study in Sweden followed 280,866 singleton men, 1475 of whom were born after a pregnancy complicated by diabetes mellitus. Maternal diabetes was associated with greater mean body mass index (BMI) in male offspring at age 18 years. This large cohort allowed comparison between siblings born before and after their mother's diagnosis of diabetes. BMI was 0.89 kg/m^2^ (95% CI 0.31–1.47) greater at age 18 years in the sibling exposed to diabetes *in utero *[17]. These findings suggest that the increase in BMI is unlikely to be due to shared lifestyle or genetic factors and that it is intrauterine exposure to hyperglycaemia that is largely responsible for the increased risk of subsequent overweight. 

A recent systematic review and meta-analysis examined offspring BMI *z*-score in childhood in relation to maternal diabetes. Analysis of data from nine studies of diverse populations showed that mean offspring BMI *z*-score was 0.28 (95% CI 0.09–0.47) higher (*P* = 0.004) for the offspring of diabetic women compared to controls born to nondiabetic women [19]. 

Offspring of diabetic women have also been shown to have higher fasting glucose levels in adolescence [18], as well as higher systolic and mean blood pressures than those born to nondiabetic women [15]. Moreover, offspring of diabetic women who are born large for gestational age (LGA) appear to be at an increased risk of adverse cardiometabolic outcomes. A longitudinal cohort study found significantly more LGA offspring of diabetic women met the National Cholesterol Education Program definition of metabolic syndrome [33] in middle childhood; 15% of offspring of diabetic women compared to 4.8% in the wider population [14].

Thus, maternal diabetes during pregnancy results in the development of many recognised cardiovascular risk factors in their progeny, with an adverse risk profile that persists into early adulthood in some studies. However, these longitudinal studies have not followed offspring of diabetic women for a sufficient time-frame to measure rates of adult cardiovascular disease outcomes such as heart attack and stroke. Increased rates of hypertension, hyperglycaemia, and overweight in young adult subjects suggest that this group is at increased risk of developing cardiac sequelae in later life, but longitudinal studies that follow the offspring of diabetic women into middle and older age are essential to quantify the clinical burden. 

## 5. Methodological Limitation of ****Current Research

Investigating the link between *in utero *exposure to diabetes mellitus and offspring cardiovascular outcomes is complicated by the long latency of disease onset, the uncertain significance of intermediate biological measurements (e.g., aortic intima-media thickness), and the multitude of confounding factors that may influence the risk of cardiovascular disease at different points in an individual's life course.

The confounding effects of other conditions linked to maternal diabetes during pregnancy also need to be carefully considered. Of primary importance, maternal obesity is a significant risk factor for the development of gestational diabetes [34] and can be directly linked to adverse infant outcomes [35]. The increased cardiovascular risk conferred on the offspring of diabetic women may largely be due to maternal overweight during pregnancy and not the diabetes *per se*. The effect of maternal diabetes on offspring BMI is partly attenuated in studies that adjust for pre-pregnancy BMI, with offspring BMI only 0.07 higher in one meta-analysis (95% CI −0.15–0.28; *P* = 0.54) [19]. A prospective longitudinal cohort study with over 4000 participants examined the relationship between pre-pregnancy BMI, gestational diabetes, and the BMI of offspring at age 16. It demonstrated maternal pre-pregnancy overweight was an independent risk factor for offspring overweight at age 16 years. The risks were highest in offspring with concomitant prenatal exposure to GDM, whereas the risks associated with GDM alone were relatively modest. Thus part of the adverse effect of maternal diabetes on offspring health appears to be accounted for by higher maternal obesity rates amongst diabetic women. There is a need for well-designed studies to examine the effects of both pathologies and their impact on offspring cardiovascular health [19]. 

Diabetes mellitus is not a homogenous disease process—T1DM, T2DM, and GDM result in exposure to dysglycaemia at different times during fetal development and hence should be studied and reported separately. Study designs also need to consider the degree of glycaemic control during pregnancy, as fetal outcomes will differ depending on the degree of exposure to hyperglycaemia. The optimal means of determining antenatal glycaemic control is uncertain. Glycosylated haemoglobin (HbA1c) can provide a crude measure of long-term control whilst neonatal cord c-peptide can provide a measure of the fetal insulin response reflecting the degree of fetal exposure to hyperglycaemia [36].

Finally, mothers and children share postnatal lifestyle factors that significantly influence the health of the offspring. In an attempt to isolate the effects of the intrauterine environment and greatly decrease the time and cost associated with longitudinal studies, surrogate imaging markers of atherosclerosis have been developed and validated. These can be utilised in the neonatal period to provide an early cardiovascular risk assessment. 

## 6. Isolating the Contribution of the *Intrauterine *Environment—Assessing Neonatal Vasculature

Maternal diabetes during pregnancy is known to affect infant vasculature. As early as 1980 Asmussen studied the umbilical vessels of infants born to women with T1DM. The umbilical vessels showed pathological changes in keeping with early atherosclerosis, including focal intimal thickening and glycogen accumulations in the cells of the intima and the media [37]. Here we summarise some of the imaging modalities available to noninvasively assess cardiovascular health. 

## 7. Carotid Intima-Media Thickness

Carotid intima-media thickness (cIMT) is a noninvasive measure of the thickness of the intima and media layers of the carotid arterial wall. In the absence of atherosclerotic plaque, B-mode ultrasound displays the vascular wall as a regular pattern that correlates with anatomical layers. The intima-media portion of this pattern is represented by the area of tissue starting at the luminal edge of the artery and ending at the boundary between the media and the adventitia. This interface is well depicted by ultrasound [38]. Carotid intima-media thickness correlates with the degree of atherosclerosis seen in the carotid artery at autopsy [39] and with findings at coronary angiography [40]. Increased cIMT is a strong predictor of future cardiovascular events in adults [41]. Carotid intima-media thickness is well established as a surrogate end point in clinical trials assessing the effects of antihypertensive and lipid lowering drugs, as it allows evaluation of changes over time [42–44]. 

Carotid intima-media thickness can be performed in the paediatric population and has been studied extensively in children and young adults with known risk factors for cardiovascular disease. Increased cIMT compared to controls is observed in children who are overweight [45], those with familial hypercholesterolaemia [46], Kawasaki disease [47], as well as in hypertensive children [48]. A trend towards a decrease in cIMT over time has been reported in a therapeutic trial of statin therapy in children with familial hypercholesterolaemia [49]. 

Ultrasound assessment of cardiovascular risk markers in childhood has many benefits. Ultrasound involves no radiation, is noninvasive, and is relatively inexpensive. However, if we wish to isolate and measure the impact of intrauterine exposure to hyperglycaemia, ultrasound measurements need to be performed in the neonatal period. Carotid intima-media thickness measurements require the subject to lie supine and remain still whilst an ultrasound probe is positioned over the carotid and measurements are taken at multiple angles [50]. Whilst some studies have successfully collected cIMT measurements in children as young as two years of age [51], measurements are logistically impossible in neonates, in part because they do not have readily accessible necks. 

## 8. Aortic Intima-Media Thickness

In neonates, the carotid artery is not accessible for ultrasound scanning; however it is possible to measure the intima-media thickness of the neonatal aorta (aortic intima-media thickness, aIMT). 

Atherosclerotic lesions develop in the aorta before they occur in the carotid; hence measuring aIMT may be not only more feasible but also a more sensitive indicator of early atherosclerosis than cIMT in neonates [52]. The Muscatine offspring study looked at both aIMT and cIMT measurements in 635 participants aged 11–34. Both aIMT and cIMT were associated with cardiovascular risk factors, and in younger participants aIMT gave information beyond that obtained from cIMT alone [52]. In particular, the aIMT was more discriminating than cIMT in high-risk subjects with diabetes and hyperlipidaemia. 

Risk factors for increased aIMT have been investigated in several paediatric studies. Aortic intima-media thickness has been found to be significantly increased in infants with intrauterine growth restriction [53] and those exposed to maternal smoking *in utero *[54]. It is also significantly increased in children with current hypercholesterolaemia and T1DM when compared to controls without these illnesses [55]. 

Only one study has investigated the contribution of maternal diabetes to offspring aIMT during the neonatal period. Increased aIMT has been demonstrated in macrosomic offspring of diabetic women when compared to controls. Macrosomic offspring of diabetic women had increased aIMT and abnormal lipid profile when compared to macrosomic offspring of controls (when adjusted for birth weight) [56]. This suggests that offspring of diabetic women are not at an increased risk simply due to their size, but due to another as yet undefined mechanism inherent in diabetic pregnancies. 

More information is needed regarding the normal and pathological range of aIMT in neonates; however aIMT has great promise as a non-invasive, relatively inexpensive, reproducible tool to quantify cardiovascular risk in infants. Further research in this area is warranted: future studies should assess women with T1DM, T2DM, and GDM separately. Maternal pre-pregnancy BMI and pregnancy weight gain should be reported, as well as degree of glycaemic control throughout pregnancy. Ideally comparisons should be made with both normal weight and overweight maternal control groups. The use of siblings, whose pregnancies were not complicated by hyperglycaemia, will be important in teasing out nongenomic factors. Finally, it would be of great interest to determine if increased aIMT measurements in this group persist into childhood and adulthood or whether postnatal influences attenuate these findings over time. 

## 9. Retinal Photography

Retinal photography is another promising option to assess the vasculature of offspring of diabetic women in the neonatal period. Atherosclerosis is a systemic vascular disease that can cause abnormalities throughout the body. Imaging of the retinal vessels has been used in many patient populations as the retina provides a unique opportunity to directly visualise a vascular bed [57]. Retinal changes may reflect preclinical changes in the coronary and cerebral microcirculations. 

In adults, retinal vessel atherosclerosis is highly correlated with atherosclerotic changes of coronary arteries seen at angiography [58]. Cohort studies of asymptomatic adult subjects demonstrated that smaller arterioles and larger venules predicted an increased risk of subsequent coronary heart disease mortality [59]. 

In the research setting, retinal photography has been successfully performed in a large number of children, including in adolescents with T2DM [60] and a cohort of over 700 schoolchildren aged 7 to 9 years in a cross-sectional study [61]. Some studies have included children as young as four years old [62], but large-scale trials have not been performed in neonates. However, the technology is already in place in many countries to image the retina of neonates. Premature infants frequently require routine examinations of their retinas to screen for retinopathy of prematurity, and many centres use digital retinal photography as part of this screening process. Retinal vessel photography is a validated research tool and may be an ideal investigation to apply to offspring of diabetic women in the neonatal period to further assess their cardiovascular risk. Potential downsides include infant discomfort [63] and lack of access to specialised equipment and operators to produce images of sufficient quality to allow robust analyses of retinal vessels. 

## 10. Echocardiography

Echocardiography has been used extensively in the neonatal period to assess the effect of *in utero* exposure to maternal diabetes. The increased rate of structural heart defects in offspring of diabetic women is clearly established [64] and will not be discussed further in this paper. Maternal diabetes is a significant risk factor for the development of hypertrophic cardiomyopathy (HCM) in the neonate, observed in 38% of cases in one series [64]. These changes appear to resolve during the first year of life [65], but there is limited evidence regarding the impact of maternal diabetes on offspring cardiac function in the long-term. One echocardiographic study demonstrated no difference in cardiac dimensions or function between children born to women with T1DM and controls in mid-childhood [66]. However, the study included only three children who experienced neonatal HCM; thus a better-powered study may have been more informative. The long term consequences of neonatal HCM remain uncertain. 

Echocardiography is a useful, non-invasive research tool that can be used across the lifespan. Further studies tracking larger numbers of offspring of diabetic women into childhood and beyond may further our understanding of the impact of maternal diabetes on the long-term cardiovascular health of their offspring. 

## 11. Electrocardiography

Electrocardiography (ECG) can also be used to assess the cardiovascular health of the offspring of diabetic women. Among adults, diabetes mellitus is associated with ECG abnormalities indicative of impaired electrogenesis. A longitudinal study of adult T1DM patients over a seven-year period showed that 18.7% of participants developed a prolonged corrected QT interval (QTc) over the course of the study [76]. This is potentially significant as QTc is predictive of all cause cardiovascular mortality, even in apparently healthy and nondiabetic individuals [77]. 

Animal models have demonstrated an association between diabetes mellitus and abnormal cardiac electrophysiology. In a diabetic rat model, isolated *ex vivo* hearts were placed under stress (using gap junction uncoupler heptanol or elevated potassium to reduce cell excitability); there was a significantly greater slowing of impulse propagation in diabetic hearts than in controls [78]. There is also impaired intercellular communication in trabeculae isolated from the ventricles of diabetic rat hearts compared to nondiabetic controls [79]. These data suggest that the diabetic heart has a substantially smaller conduction reserve than the nondiabetic heart, rendering it vulnerable to conduction disturbances and arrhythmias caused by physiological and pathological challenges such as ischaemia or exercise [78]. 

Transient disturbances in glucose metabolism may also result in quantifiable ECG changes. In healthy adults, hyperinsulinaemia-induced hypoglycaemia can prolong the QTc interval and decrease T-wave area and amplitude [80]. This ECG-based assessment may be of particular relevance in studies of the offspring of diabetic women during the neonatal period, as these infants are at a higher risk of hyperinsulinaemia-induced hypoglycaemia than the general population. 

Studies examining ECG changes in the offspring of diabetic women are, however, limited. Bacharova et al. performed ECGs on 109 offspring of diabetic women as well as 81 control infants. A modest but statistically significant leftward shift of electrical axis was observed in the offspring of diabetic women [68]. Infants of diabetic women also appear to be less able physiologically to respond to the stress of labour; on fetal ECG, these infants were more likely to demonstrate ST depression than infants of control women [69]. 

Electrocardiography is a non-invasive, inexpensive, and very well-established method of assessing cardiovascular health. ECG is therefore a useful potential adjunct investigation to assess the cardiovascular health of infants of diabetic mothers. Future research should aim to recruit large cohorts of infants born to diabetic women and follow them through childhood and into adulthood to determine the effect of intrauterine exposure to hyperglycaemia on future cardiovascular health. Electrocardiographs should be reviewed looking for signs that are seen in diabetic patients—sinus tachycardia, long QTc, QT dispersion, changes in heart rate variability, ST-T changes, and left ventricular hypertrophy [81].

## 12. Other Modalities

A number of other modalities, both conventional and novel, can be used in older offspring of diabetic women. These and the modalities discussed previously are summarised in Table 1.

## 13. Clinical Relevance and Future Directions

Exposure to diabetes during pregnancy has diverse negative effects on the cardiovascular health of the developing child. Some of these effects may be related to coexisting maternal overweight/obesity and possibly dyslipidaemia, which are often correlated with diabetes. The degree of glycaemic control during pregnancy and the timing of the onset of hyperglycaemia are likely to influence the impact on fetal development. 

Longitudinal studies have demonstrated that offspring of diabetic women are at an increased risk of developing a number of cardiovascular risk factors including hypertension, impaired glucose tolerance, dyslipidaemia, and overweight/obesity. Some of these negative health outcomes may be due to genetic or postnatal lifestyle exposures, but the evidence to date indicates that *in utero *exposure to hyperglycaemia does play a significant role. 

Novel imaging techniques applied during the newborn period will allow a more precise assessment of the effect of the intrauterine environment. Further research is needed in this domain to determine how aIMT findings in neonates translate into cardiovascular disease in adults. Offspring of diabetic women may need to be considered a high-risk group for more intensive cardiovascular monitoring in the future. Importantly, in the face of the obesity “epidemic” and rising rates of diabetes in pregnancy, increased focus on initiating disease prevention *in utero* may have dramatic effects on population-level adult cardiovascular health outcomes.

## Figures and Tables

**Table 1:** Assessment of cardiovascular health in prepubescent offspring of diabetic women.

Modality	Age	Method	Limitations	Evidence in children of diabetics
Blood pressure	From birth, but difficult to perform reliably in infants	Oscillometric (most common) or auscultatory measurement of blood pressure with sphygmomanometer. Subjects need to be completely relaxed, in a uniform position (e.g., sitting) and uniform site of measurement (e.g., right arm). Cuff needs to be correctly sized. Minimum of two measurements.	May be difficult to obtain “resting” blood pressure in young children—as the cuffs can be uncomfortable and cause distress.	Large cohort study of school children looked at subgroup of offspring of diabetic women and found no difference in systolic or diastolic blood pressure [67]. Other studies have demonstrated higher systolic and mean blood pressures in offspring of diabetic women than controls [15].

Electrocardiography (ECG)	All ages—can be performed *in utero *	Leads are placed on chest wall in standard locations. Electrical impulses are transmitted to ECG machine and ECG tracing is produced.	ECG most accurate if patient remains still during reading—can be difficult for young children. Requires the subject to be at a comparable level of exertion (e.g., at rest).	Infants of diabetic women demonstrate leftward axis deviation when compared to controls [68]. Fetuses of diabetic women demonstrate significantly more ST depression on fetal ECG during labour than infants of control women [69].

Vessel structure				

Carotid artery intima-media thickness (cIMT)	From 5 years of age	High-resolution B-mode ultrasonography. Measure thickness of intima-media of posterior wall of carotid. In adults correlates to atherosclerosis at autopsy [39] and coronary angiography [40] as well as predictor of future cardiovascular events [41].	Need to be able to access the neck over the carotid area [50]. Has been used in children >5 years old [70], technically difficult in younger children. Skilled operator required.	Although cIMT has been used to follow up mothers with gestational diabetes [71, 72], currently no studies in offspring of diabetic women.

Aortic intima-media thickness (aIMT)	From birth/*in utero *	High-resolution B-mode ultrasonography. Measure thickness of intima-media of posterior wall of abdominal aorta. Good correlation with cIMT [55].	Most easily performed on the settled infant/child. Yet to be correlated with long-term disease risk.	Increased aIMT found in offspring of diabetic women (additive effect with maternal obesity) [10].

Retinal vessel imaging	From birth (pupillary dilatation required) and from school age without dilatation	45° digital retinal photography following pupil dilatation [62]. Method has been used to evaluate retinopathy of prematurity in neonates.	Requires pupil dilation. May be uncomfortable for infants [63].	No current studies in offspring of diabetic women. Two large unselected cohort studies in children underway in Australia and Singapore.

Endothelial function				

Pulse wave velocity (PWV)	From 5 years of age	Peripheral pulse wave form measured at the radial artery, carotid and femoral artery in order to calculate central aortic pressure as well as carotid-femoral pulse wave velocity.	Would be limited by cooperation and physical access in younger children.	One study looking at offspring of diabetic women at ~15 years found positive correlation between GDM (umbilical cord c-peptide, insulin) and arterial stiffness [73].

Brachial artery dilatation	Older children (~10 years and above)	B-mode ultrasound—assessing dilator response of brachial artery to endothelial dependent and nondependent factors. First stage—pneumatic cuff suprasystolic for 5 minutes then deflates and measures brachial dilatation. Second stage—GTN given orally and measures brachial dilatation.	Poorly tolerated by children (both time taken and cuff discomfort). Invasive (administering GTN orally). Need to be fasting.	Studies in women with GDM—one study found no change [74]. No studies in offspring of diabetic women.

Endopat	Older children (~10 years and above)	Similar to brachial artery dilatation. Endopat apparatus on finger—measuring pulse wave. Measured at rest, then with pneumatic cuff inflated to 60 mmHg or 200 mmHg for one minute, then measured following deflation [75].	Takes 15 minutes. Need to be fasting. May be difficult to tolerate in children (especially pneumatic cuff over systolic BP). Expensive.	No data in either diabetic women or their offspring.

Cardiac function				

Echocardiography	From birth/*in utero *	2-dimensional, M mode and Doppler ultrasonography of the heart.	Requires specialist paediatric echocardiographer/ sonographer with experience in interpreting paediatric findings.	Increased risk of HCM in offspring of diabetic women [64]. Resolution of changes by first year of life [65]. No studies following offspring of diabetic women into adulthood.
